# Shaping Student Relationships: The Role of Personality in Early Childhood Pre-Service Teachers

**DOI:** 10.3390/bs14090778

**Published:** 2024-09-05

**Authors:** Seda Ata, İlayda Kimzan

**Affiliations:** Department of Early Childhood Education, Muğla Sıtkı Koçman University, Muğla 48121, Türkiye; sedaata@mu.edu.tr

**Keywords:** student–teacher relationships, personality, preschool education, pre-service teachers, temperament

## Abstract

The purpose of this explanatory sequential mixed-methods study is to explain teacher–student relationships in preschool classrooms in terms of the child’s temperament and the pre-service preschool teachers’ personalities. The study was conducted using a sequential exploratory mixed-methods design. Since both quantitative and qualitative data were obtained, sampling was carried out in two stages: quantitative random stratified sampling, and qualitative purposive sampling. Quantitative data were obtained from 126 pre-service teachers. The qualitative study group consisted of 18 pre-service teachers. Quantitative data were collected using the Student–Teacher Relationship Scale-Short Form, the Short Temperament Scale for Children (STSC), and the Five Factor Personality Inventory (FPI). Qualitative data were obtained from interviews with 18 teachers. The findings revealed that the student–teacher relationship can be explained by adult and child characteristics. In addition, pre-service teachers’ perceptions of the student–teacher relationship are explained by adult characteristics much more than pre-service teachers’ perceptions.

## 1. Introduction

The relationship between students and teachers, defined as intimacy and conflict during the preschool years, plays a significant role in children’s development [1]. It is possible to argue that attachment theory has an impact on the significance of these relationships. According to attachment theory, the nature of the bond children form with their caregivers shapes attachment patterns in the children. Attachment theory is supported by the research of Bowlby [2], Ainsworth [3], and Sroufe et al. [4]. In this theory, a child’s relationship with their preschool teacher is critical [5,6]. Children’s emotional relationships with their teachers support their short- and long-term learning and development by providing the protective and supportive environments they need [7]. Positive student–teacher relationships are a vital resource for children, which make significant contributions to their development. When student–teacher relationships are characterized by positivity, warmth, and the absence of conflict, the child can use the relationship with their teachers to safely explore the school environment [8,9]. Children will continue to seek support from teachers in subsequent relationships, establishing or hoping for a similarly positive and supportive relationship with them. Furthermore, we can assume that a close student–teacher relationship fosters not only a positive relationship with their teachers, but also commitment, as children tend to engage more in activities with teachers who are responsive to their needs and interests [10,11]. Intimacy reflects the degree of warmth and open communication in the student–teacher relationship. Close student–teacher relationships help children feel emotionally secure, which supports their exploration in the classroom and motivates them to participate in more educational activities [12].

Research has shown that student–teacher conflict is a significant barrier to children’s active participation in the classroom [1]. This conflict can not only reduce students’ engagement in classroom activities but can also lead to increased misbehaviors [10] and decreased cooperation [13]. Conflictual student–teacher relationships negatively impact a child’s sense of belonging, perceived competence, and ultimately, participation [14]. This can hinder students’ ability to develop positive relationships with teachers, potentially leading to conflict and a lack of intimacy in future teacher interactions [15,16,17].

In contrast, positive relationships with teachers are a crucial factor that promotes children’s active participation in the classroom [18]. In this context, it is essential for teachers to avoid conflict with students and strive to build close and supportive relationships with them, as this is highly beneficial for students’ academic and social development. Once more, within the confines of this particular theory, a child’s gender (e.g., [19]), their temperamental tendencies (e.g., [20]), as well as their subsequent social–emotional progress (e.g., [21]) and academic accomplishments (e.g., [22]), alongside self-regulation (e.g., [23]), are also impacted.

The student–teacher relationship in early childhood is significantly shaped by the active participation of children, who play a crucial role in determining the nature of these interactions. Children’s personalities, behaviors, and engagement levels heavily influence how teachers perceive and respond to them, making the child a central figure in this dynamic teacher–student relationship. [24]. Paes et al. [25] further highlight the long-term effects of these relationships, showing that positive interactions between students and teachers in early childhood can lead to improved academic achievement later on. Similarly, Šumatić et al. [26] emphasize the importance of considering both child and teacher characteristics, along with the classroom environment, in shaping the quality of student–teacher relationships. They note that these relationships are influenced by various external factors, such as the cultural and educational context in which they develop.

On the other hand, Embacher and Smidt [27] explore how child personality types can impact the closeness and conflict experienced in teacher–child relationships, demonstrating that certain personality traits may lead to stronger or more challenging interactions. Wang et al. [28] extend this by investigating the role of teacher traits, such as mindfulness and emotional intelligence, in encouraging positive student–teacher relationships, emphasizing that teachers’ own characteristics also significantly shape the relational quality.

However, this relationship is not solely child-driven. Research shows that the effects are bidirectional, with both student and teacher characteristics shaping the quality of their interactions [29,30]. Thus, the student–teacher relationship is a complex, reciprocal process influenced by a range of factors from both sides, as well as the broader classroom environment [31]. Numerous factors influence the quality of this crucial relationship, and recent studies have highlighted the significance of child characteristics in shaping teachers’ and children’s perceptions of the quality of the student–teacher relationship. Among these features, we will explain temperament, personality, and the teaching practicum in detail below.

### 1.1. Personality

Various studies [32,33] assume that a teacher’s personality influences the development of schemas about how teaching should be in the classroom and its overall performance. For example, Bullock, Coplan, and Bosacki [34] reported that preschool teachers’ openness to experience and extroversion predicted classroom management self-efficacy (when controlling for teacher years of experience). Research linking teachers’ personalities to attitudes and strategies with students is limited [35]. However, teachers generally have a high average in extraversion [36], and teachers’ extraversion and openness predicted their sense of efficacy when working in childcare and preschool classrooms [34].

Surprisingly little research has examined the role of pre-service teachers’ personalities in competency, attitude, and behavior management strategies. The sample in this study consisted of pre-service teachers (i.e., who are in the process of completing the formal teacher education program). Research suggests that the relatively little teaching experience of pre-service teachers may strongly relate their attitudes and reactions in the classroom to their characteristics, such as personality traits [37,38].

Teachers’ statements and perspectives generally reshape research on the student–teacher relationship. In this context, research findings in related literature suggest that teachers have the ability to reflect their perspectives in these relationships. For instance, scholars believe that the ethnic origins and gender of the children influence the teachers’ evaluations of them [21,39,40].

Corbin and colleagues’ [41] study demonstrated that teachers’ stress levels and self-efficacy beliefs directly influence the quality of student relationships. Stressed or exhausted teachers are more likely to experience conflicted relationships with children, which shapes their responses to classroom relationships [20]. Embacher and Smidt [27] found that teachers’ professional competencies and job satisfaction are important factors influencing the quality of teacher–child relationships. Despite the relatively smaller number of variables related to teachers, studies on this topic show that many psychological characteristics of teachers are an important component of the student–teacher relationship (see also [28]).

Previous research, focusing on the interactive nature of the student–teacher relationship, has primarily emphasized the role of children’s traits, particularly internalizing and externalizing behaviors, in shaping the quality of the relationship [42]. However, the impact of teachers’ own personalities on this interactive dynamic has been less extensively explored. Understanding the relationships between pre-service teachers’ personality traits and their attitudes and reactions will provide insight into the decision-making processes of pre-service teachers regarding related behaviors and, especially, examines the importance of teacher candidates’ relationships with children; their perceptions of these relationships will make significant contributions.

### 1.2. Teaching Practicum

A qualified preschool teacher’s curriculum knowledge enables them to create a disciplined learning environment [43], make learning meaningful [44,45], and be supportive of children. Howes et al. [46] expect excellent performance in areas such as conducting interactions. In the realization of these expectations, pre-service practices, in which teacher candidates transform their theoretical knowledge into practice, play an important role (e.g., [47,48]). Practicum experiences not only provide pre-service teachers with an opportunity to put theory into practice, but they also help teachers adopt a holistic view of teaching (e.g., understand the workings of schools and classrooms, become familiar with the school environment and what it means to be a teacher) before entering the workplace [49]. Therefore, pre-service teachers are empowered by their professional knowledge and realization of their strengths and weaknesses in both theoretical and practical knowledge.

Practicum experiences have been defined as one of the most important steps to becoming a teacher, as well as one of the most important experiences in teacher education programs [50,51]. Classroom-based opportunities conducted under the supervision of a lead classroom teacher (i.e., the collaborating teacher) provide experiences for trainees to develop an “experience-based understanding” of children’s learning and appropriate teaching strategies under the guidance of a collaborating teacher [52]. Teacher training programs enable pre-service teachers to develop into effective teachers by expanding their experiences with classroom practices and providing a strong infrastructure through various courses. To obtain a degree as a preschool teacher in Turkey, a total of 240 ECTS education credits are required in the preschool education department of the Faculty of Education. These undergraduate programs include Teaching Practice I and II as required courses. Within the scope of these courses, which occur in the last year of the 4-year undergraduate program, prospective teachers attend preschool education institutions.

The aim of this study is to determine whether pre-service teachers’ statements about their relationships with children during their internship practices, in which they increase their professional knowledge and experience, are more affected by their own personality traits or children’s temperaments. In this study, prospective teachers’ perspectives on their relationships with children were analyzed in a multidimensional way.

## 2. Methods

We employed the explanatory sequential mixed-methods design in this study. Mixed-methods research design is a procedure for collecting and analyzing data produced by multiple methods, usually both quantitative and qualitative methods, in which data are mixed or integrated at some stage of the research process to gain a better understanding of the data [53]. The explanatory sequential mixed-methods design usually consists of two distinct phases. The researcher first collects and analyzes quantitative data, followed by a qualitative data collection and analysis phase to help refine, explain, enhance, or refine the initial quantitative results [54].

The purpose of this explanatory sequential mixed-methods study was to explain teacher–student relationships in preschool programs in terms of the child’s temperament and the pre-service preschool teachers’ personalities. In mixed-methods research, the investigator uses both quantitative and qualitative approaches to collect and analyze data, integrate the findings, and draw inferences [55].

### 2.1. Participation

The sample for this study consists of two different groups. The first group of participants consisted of pre-service preschool teachers (N = 126) and the second group of participants consisted of preschool students (N = 252; 120 girls and 132 boys aged between 36 and 60 months; M = 42.8, SD = 7.92). All of the participants were working in 12 (twelve) preschool education centers in Muğla and Istanbul, working with preschool children. The convenience sampling method selected the study group.

The participants consisted of 126 pre-service preschool teachers between the ages of 21 and 25 (M = 23.46, SD = 4.51) enrolled in teacher training programs (4th year of the Early Childhood Education bachelor’s degree) at two universities in Turkey. A total of 80 pre-service teachers from the university in Istanbul and 46 pre-service teachers from the university in Muğla participated in the study. The numerical difference is due to Istanbul’s larger university and student population.

Except for elective courses, the current program in the Early Childhood Education bachelor’s degree at Muğla and Istanbul universities is exactly the same in terms of basic field education. Therefore, all pre-service teachers underwent the same educational processes and successfully completed the practicum II course experience.

After practicum I, which is the first formal field experience, the practicum II course requires students to spend at least 6 h a week for 12 weeks in practicum schools. During the practicum I course, pre-service teachers were required to attend preschool education centers for 12 weeks. After taking the practicum II course in the spring semester, they should start in the spring semester. In this study, 126 pre-service teachers were asked to complete data collection tools for students they had known for at least 7 months (20 weeks). Each pre-service teacher takes on the role of a teacher for 6 h in the classroom every week for 24 weeks and implements a pre-planned daily education flow with children. There are at least four activities in each daily education flow. Additionally, pre-service teachers lead free play times and meal/rest times. All of these activities encompass the types of activities included in the preschool education program, such as art, Turkish language, mathematics, drama, science, music, introduction to literacy, games, and field trips, and they are distributed in close proximity to each other. Therefore, pre-service teachers and children interacted intensively with each other and developed teacher–student relationships. 

The 18 pre-service teachers with whom the qualitative interviews were conducted were selected through purposive sampling. This selection was based on the results obtained after the pre-service teachers completed the Five Factor Personality Inventory, and the same number of personality types were randomly selected. As a result, 18 pre-service teachers who could serve the purpose with maximum diversification were selected as participants.

### 2.2. Procedure

We primarily collected the data between March and May 2022. We collected survey data from all participants in the late spring. We clearly explained that participation in the research process was based on eagerness, and the participants can quit the process when they want to. Initially, we introduced the research intentions to about 126 pre-service teachers who agreed to participate.

We contacted 15 preschool centers by phone, and 12 of the 15 directors agreed to make an appointment and listen to the working process. The researchers first informed the principals about the study during their visits. After their approval, the researcher contacted the teachers and provided information about the study. The researcher emphasized the importance of voluntary participation in this context. In addition, measurement tools were introduced. Pre-service teachers evaluated the student–teacher relationship form, while mothers evaluated the child temperament form.

We asked the pre-service preschool teachers to complete questionnaires about themselves and their students. Due to time constraints, the pre-service teachers completed questionnaires for a smaller number of students who were randomly selected (approximately 1–3 students).

The pre-service teachers spent about 15–20 min to fill out a paper questionnaire that included demographic information for about 2 students and questions about student–teacher relationships with randomly selected children. In addition, 18 pre-service teachers selected through purposive sampling were interviewed about their relationships with these children.

Preschool teachers delivered the temperament scale to the families. The preschool teachers asked the mothers to fill in the forms about their children’s temperament. Information regarding the temperament traits of the children was collected from the mothers, who had the best and most extensive opportunity to observe their children from birth. Each mother completed the temperament assessment tool for her own child, based on the understanding that the most accurate information about the children’s behavior and characteristics would come from those who know them best—their parents—rather than from the educators.

### 2.3. Measures

Demographics: Pre-service teachers and mothers of preschool children completed a demographic questionnaire including age and gender information. We administered the following instruments to pre-service teachers and mothers of preschool children.

#### 2.3.1. Student–Teacher Relationship Scale-Short Form (STRS-SF)

The teachers’ judgments of the quality of their interactions with the participating children were assessed using the STRS-SF. Pianta [1] produced the STRS-SF, which consists of 15 items out of the original 28 items and use a Likert-type format. This measuring instrument consists of two subscales: intimacy and conflict. ‘I share an affectionate, warm relationship with this child’ is an example of an item from the closeness subscale, which comprises eight items and assesses teachers’ views of warmth and bonding with a specific child. The conflict subscale comprises seven items and assesses teachers’ perceived disagreement with a specific child (e.g., This child and I always seem to be struggling with each other). Participants were instructed to rate the extent to which each statement reflected their relationship with the student on a 5-point scale (1 = ‘Does not apply’ and 5 = ‘Definitely applies’), where higher scores indicated greater intimacy or conflict. A validation of the STRS-SF was conducted using Turkish preschool children [56]. The Turkish version of the study involved 22 institutions, serving 830 students and 165 teachers who participated by completing the scales. The reliability analysis revealed an internal consistency coefficient of 0.82 for the total score (Cronbach alpha). Subscale evaluation yielded an internal consistency coefficient of 0.84 for the conflict subscale and 0.76 for the closeness subscale. We calculated test-retest reliability coefficients for the conflict subscale at 0.87, the closeness subscale at 0.83, and the total score at 0.83. The CFI (comparative fit index) value was 0.97, the GFI (goodness of fit index) value was 0.96, the NFI (normed fit index) value was 0.96, and the RMSEA (root mean square error of approximation) was 0.053 (RMSEA 90% confidence interval limits were 0.046; 0.060). When the fit statistics were examined in which all-model data fit was evaluated, it was found that the scale items with 15 indicator variables showed a high level of fit in general. As a result, confirmatory construct validity of the teacher–student relationship scale was achieved. The findings revealed the preservation of the two-factor structure in the original short form of the scale when we forced the 15 items in the scale into a two-factor structure. Furthermore, the confirmatory factor analysis results demonstrate the achievement of the confirmatory construct validity in the student–teacher relationship scale.

The original validation of the STRS did not explicitly include pre-service teachers as part of its sample. Given this, the current study carefully considered the psychometric properties of the STRS-SF within the context of pre-service teachers. Specifically, the reliability, as indicated by Cronbach’s alpha, and the validity of the scale were assessed and reported. This thorough examination ensures that the findings are both reliable and valid when applied to the pre-service teacher population, confirming the scale’s appropriateness for this particular context.

#### 2.3.2. Child’s Temperament Scale

Prior, Sanson, and Oberklaid established the Short Temperament Scale for Children (STSC) [57], which was then modified for use in Turkish by Yagmurlu and Sanson [58]. The STSC is assessed using a 6-point Likert response scale. The assessment has 30 items distributed over four dimensions: Reactivity, Persistence, Approach, and Rhythmicity. The STSC has 30 items, with each behavior being assessed using a 6-point scale.The scale taps four temperamental dimensions: Reactivity (e.g., ‘When upset or annoyed with a task, my child throws it down, cries, slams doors, etc.’), Persistence (e.g., ‘My child likes to complete one task or activity before going on to the next’), Approach (e.g., ‘My child is shy when first meeting new children’), and Rhythmicity (e.g., ‘My child asks for or takes a snack about the same time each day’). A high score on each axis corresponds to reactive, persistent, withdrawing, and arrhythmic temperamental characteristics, respectively. The analysis confirmed strong internal consistency for all temperament dimensions. The Cronbach alpha coefficients for the reactivity subscale, approach, persistence, and rhythmicity subscales were 0.69, 0.79, 0.75, and 0.63, respectively. Being of little theoretical significance, the present study did not analyze rhythmicity.

#### 2.3.3. Five Factor Personality Inventory

In this study, the 10-item short form of the Five Factor Personality Inventory (FPI) developed by Rammstedt and John was used [59]. The scale is used to determine the personality traits of individuals. Horzum, Ayas, and Padır [60] carried out translation studies of the scale into Turkish. It is a 5-point Likert-type (never–always) 10-item scale. Researchers conducted validity and reliability studies with three distinct groups. In the first study group, four lecturers were selected who are experts in the field of psychological counseling and guidance, who studied in the field of personality constructs, and who have a good command of English. The second study group was composed of 31 students studying English language teaching for linguistic equivalence. The third study group consisted of 218 high school students who were selected to analyze the factorial and construct validity of the scale. The findings from the scale’s linguistic equivalence study revealed that the correlation between the Turkish and English original forms was quite high. The results of the exploratory and confirmatory factor analysis of the scale, the construct validity, and the findings obtained for the scale’s reliability showed that the scale is a reliable measurement tool in Turkish culture. In the scale adaptation study, it was concluded that the FPI consists of five factors: Extraversion, Agreeableness, Conscientiousness, Openness, and Neuroticism. In the adaptation study of the scale, the internal consistency coefficient for Extraversion was 0.88, while that for Agreeableness was 0.81, for Conscientiousness was 0.90, for Openness to Experience was 0.84, and for Emotional instability was 0.85. Items 1, 3, 4, 5, and 7 of the scale are reversely scored. The personality trait related to the sub-dimension with the highest score by looking at the scores of the individuals from each sub-scale is called the basic personality trait of the individual.

#### 2.3.4. Analysis

The data collected through three different scales were first subjected to a normality test. Then, the descriptive statistics of the data were calculated, and the necessary analyses were carried out by constructing a regression model. IBM SPSS Statistics for Windows 21.0 (IBM Corp., Armonk, NY, USA) was used for data analysis. Means and standard deviations were calculated to describe the characteristics of the students and the group as a whole. Pearson correlation analysis was used to find the relationships between independent and dependent variables. Stepwise multiple regression analysis was used to determine how teachers’ personality traits and students’ gender and temperament affect student–teacher relationships. The data met the assumptions of normality, and the unstandardized residual skewness is 0.58. The kurtosis is 1.18, which is within the typically recommended skewness and kurtosis guidelines of <2.00 (e.g., [61]). Furthermore, multicollinearity between independent variables was investigated by examining variance inflation factors (VIF), which are all less than 10, indicating that multicollinearity is not an issue. The Mahalanobis distance showed that there were no outliers in the data.

### 2.4. Qualitative Data, Procedure, and Analysis

The qualitative data in this study were collected via a semi-structured interview form developed by the researchers in the context of student–teacher relationships in preschool settings. In this study, using a semi-structured interview form, we aimed to determine the basic questions that the researcher should ask during the interview, and provide new ideas with additional questions to be asked according to the answers to be given by the participant [62]. The quantitative analysis revealed three personality traits based on significant sub-variables. For the qualitative part, interviews were conducted with individuals selected according to these three different personality types to represent these traits (Table 1).

Semi-structured interviews with a total of 18 pre-service teachers were used to collect the data in April 2022. The semi-structured interviews used nine researcher-developed questions relevant to the study. Open-ended questions were developed by the researchers. In addition, for some open-ended questions, we identified exploratory questions that would allow us to obtain more detailed information. After the questions were organized in logical order, these inquiries underwent an initial review by field experts, leading to revisions based on their feedback. Then, we incorporated pre-service teacher perspectives and optimized the final version for clarity. The interview methodology encompassed inquiries regarding the participants’ overall perspectives on the teacher–student relationship, their strategies for establishing relationships, their comprehension of their assigned responsibilities in the classroom, and their opinions on classroom authority. Then, we conducted one-on-one interviews with these volunteers in the appropriate classrooms at the universities. At this time, the researcher explained why they wished to use audio recording and sought the volunteers’ permission to do so. All of the participants consented to audio recording, and the interviews lasted between 20 and 35 min.

The researchers transcribed all recorded interviews. The researcher and a second coder, who had experience in qualitative data analysis, then read the transcripts separately several times and coded the data. This study employed two techniques for qualitative data analysis: word lists and keywords in context [63]. As suggested by Bernard and Ryan [63], the researcher and the second coder tried to identify words and phrases in the transcripts that were particularly relevant to the sub-topic of the study.

**Figure 1:**
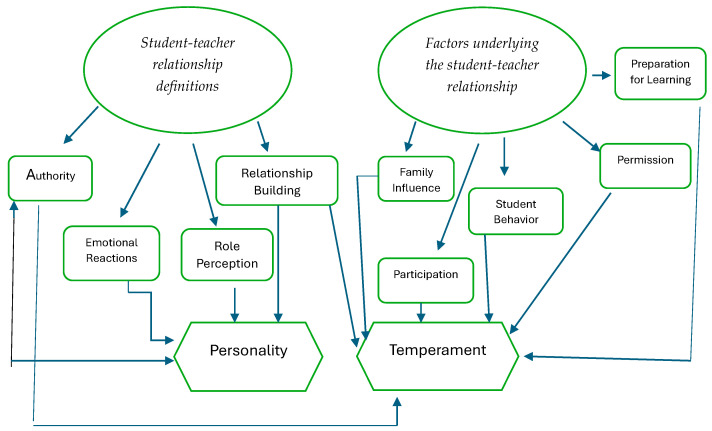
Thematic map.

In this study, thematic analysis was used to analyze qualitative data. The thematic analysis approach is used to identify patterns in the data, organize them into the fewest dimensions and describe them in depth [64]. In this analysis, themes or patterns can be obtained in two main ways: inductive and deductive. Researchers using the deductive coding approach created a codebook [65]. First, one of the researchers selected specific concepts based on the theoretical framework. This is in line with Creswell’s [65] suggestion to use pre-existing codes that guide the research process. Preliminary codes were created based on these concepts. Codes such as perception of authority, family influence, emotional reactions, and role perception were identified. Code definitions were created in line with the emerging codes. The definitions and codes created a reference point for the coding process. We analyzed the data set and started the coding process. At the end of the process, we compared the codes from two different coders, and in some cases, we reached consensus on the codes. As a result, based on these codes, we identified two primary themes. The relationship between themes and codes is shown in Figure 1. With the benefit of creating a deductive codebook during the coding process, we did not find any significant inconsistencies between the researchers, which did not lead to the calculation of inter-coder reliability.

## 3. Results

The findings presentation consists of three stages. Phase 1 involved the explanation of the quantitative data from the research, including descriptive statistics, correlation coefficients, and multiple linear regression. Phase 2 involved thematic analysis to analyze the interviews conducted after the purposeful sampling, which was chosen based on the results of the quantitative data. The results were broken down into two main themes. In Phase 3, qualitative data and teachers’ personal perceptions of classroom relationships reinforced the significance of these three personality dimensions in quantitative analyses, shaping student–teacher relationships. Simultaneously, we examined the role of children’s temperament traits in these relationships and discussed their dynamics in a broader context.

### 3.1. Phase 1

Table 2 and Table 3 provide the means and standard deviations of the variables.

Table 2 shows descriptive statistics for the reported variables. Closeness in student–teacher relationships received the highest ratings by pre-service teachers (M = 3.00, SD = 1.609). Additionally, conflict in student–teacher relationships received a lower rating than closeness. Table 2 also shows the correlations between predictor and outcome variables. Agreeableness was positively correlated with pre-service teachers’ perceptions of closeness in relationships with students. Neuroticism in pre-service teachers and reactivity in preschoolers were positively associated with pre-service teachers’ perceptions of conflict in relationships with students. The results obtained from the regression analysis of pre-service teachers’ perceptions of teacher–student relationships on teachers’ personalities and children’s temperaments are detailed in Table 3.

#### 3.1.1. Closeness

As a result of the analysis, a significant regression model was established, (F(9,116) = 0.790, *p* > 0.05), with the coefficient of determination (R Square) being 0.15. The model indicated that 15% of the variance in the independent variable was explained by the independent variables. Agreeableness predicted closeness positively and insignificantly, β = 0.653, t(116) = 1.646, *p* > 0.05, pr2 = 0.2. Neuroticism predicted conflict negatively and insignificantly, β = −0.168, t(116) = −0.520, *p* > 0.05, pr2 = 0.2. The other characteristics of the pre-service teachers’ personalities and children’s temperaments did not explain a significant percentage of the variance in the closeness of student–teacher relationships.

#### 3.1.2. Conflict

As a result of the analysis, a significant regression model was established (F(9,116) = 0.792, *p* > 0.05), with the coefficient of determination (R square) being 0.32. The model indicated that 32% of the variance in the independent variable was explained by the independent variables. Neuroticism predicted conflict positively and insignificantly, β = 0.495, t(116) = 1.606, *p* > 0.05, pr2 = 0.5. Reactivity predicted conflict positively and insignificantly, β = 0.593, t(116) = 1.827, *p* > 0.05, pr2 = 0.5. The other characteristics of the pre-service teachers’ personalities and children’s temperaments did not explain a significant percentage of the variance in the conflict of student–teacher relationships.

### 3.2. Phase 2

We selected 18 pre-service teachers, based on the quantitative analysis findings, and conducted in-depth interviews with these participants. The main goal of the interviews was to reveal the pre-service teachers’ views on the teacher–student relationship in greater detail. These participants were selected based on the personality traits of the prospective teachers and their voluntary participation. We grouped the codes that emerged from the analysis under two main themes. The first is a definition of conflict and closeness in relation to the student–teacher relationship. The second one is the reasons underlying conflict and closeness in the context of student–teacher relationships.

#### 3.2.1. Student–Teacher Relationship Definitions

Pre-service teachers’ definitions of intimacy focused more on the different levels of intimacy established individually. Conflict definitions were more common for pre-service teachers. Some pre-service teachers stated that they did not follow the instructions, exhibited undesirable behaviors in the classroom, and that there was disharmony in the definitions of conflict. Especially for pre-service teachers, the code of not accepting authority came to the fore.

A pre-service teacher with a high level of agreeableness used the following expressions to describe her relationship with children: “*A different level of closeness with children can be seen among us trainee teachers… It is important to be loving and try to interact with children in some way. The main reason for this is the impact we have on children. While some of us prefer to be more like an authority figure, some of us create the image of a teacher who tries to have a pleasant time with children*” (high level of agreeableness, pre-service teacher).

When the teacher–student relationship was assessed through the lens of pre-service teachers, characterized by elevated levels of openness and compatibility, certain opinions articulated by them are delineated earlier. It is possible that pre-service teachers who demonstrate openness and compatibility in their dispositions attempt to cultivate their interactions from a heightened emotional vantage point. These efforts imply that they are attempting to establish a connection with students through activities perceived as more enjoyable, compassionate, and pleasing.

A pre-service teacher with a high level of neuroticism made the following comments about how being an authority or not being an authority affected her communication with children: “*Some children may not take the trainee teacher seriously at first. For example, not being taken seriously makes me angry right away. I do not think I can establish a close relationship with these children. They see us more as their older brothers or sisters who come to their classes. I get frustrated at this situation. On days like these, I struggle greatly to assume the role of a teacher as a pre-service teacher*”.

Based on pre-service teachers’ perspectives, certain scenarios emerged concerning the structuring of student–teacher relationships. These data led to a significant focus on the efforts of educators with neurotic personality traits to assert their authority. It is plausible to argue that pre-service teachers endeavor to delineate their respective roles through the exercise of authority. They highlighted instances where they grappled with the dilemma of potentially compromising their authority while addressing the individual needs of students or when actively enforcing classroom regulations to engage in class activities. This dilemma manifests in their interactions with students.

#### 3.2.2. Factors Underlying the Student–Teacher Relationship

While emphasizing the importance of the pre-service teachers’ personal characteristics and communication styles in the student–teacher relationship, they also stated that the differences in these relationships, which stem from the students’ individual and family structures, significantly influence the student–teacher relationship. The pre-service teachers stated that the crowded class size affects the student–teacher relationship, which operates on different levels.

One of the pre-service teachers with a high level of neuroticism expressed his views on the overcrowding of the classroom population and its effect on the teacher–student relationship. The teacher candidate articulated his views as follows: “*Besides, a classroom teacher can’t necessarily care for 25 children at once; we deal with the children more one-on-one, so the children tell everything and see us closer to them. Of course, this does not apply to all children. Not all teachers are equally sympathetic to the children. The underlying reasons for this may be their temperament, the teacher’s or the teachers’ worldviews, their perspectives on life, the living conditions they were born and raised in, their family structure, and the attitudes that their families apply to the teacher candidates*” (high level of neuroticism, pre-service teacher). As can be seen, this candidate emphasized both the one-to-one interaction in teachers’ relationships with children and the importance of adults’ temperaments and life experiences.

There is a strong understanding that the bond developed with the family is very effective on the basis of children’s relationships with their teachers and that the teacher–student relationship is built on this. At the same time, the effects of environmental conditions and life experience can be handled in a similar way.

In general, pre-service teachers think that the time spent with children affects their relationships with them. Therefore, they think that the relationships between teachers and children differ at this point. They believe that spending more time together facilitates communication, and even if there is a negative conflict situation, it can be resolved more easily. They also believed that this was directly related to establishing authority. “*I believe that when there is a conflict in the classroom, the classroom teachers and the children can establish a closer relationship and resolve things more quickly”. “Some children may not take the trainee teacher seriously at first. I don’t believe that trainee teacher candidates share the same level of closeness or conflict with the children*” (high level of agreeableness, pre-service teacher). Another notable statement from pre-service teachers is that children’s willingness to participate in activities significantly influences their relationship with the teacher. Pre-service teachers remarked that children who eagerly engage in classroom activities often do so because they seek more interaction and connection with the teacher. For example, “*When my instructions catch their attention, it is clear that children who are enthusiastic about participating in activities are also the ones who want to see us more in the classroom. This eagerness helps foster a strong bond and makes classroom management smoother*” (high level of openness, pre-service teacher).

### 3.3. Phase 3

#### 3.3.1. High Neuroticism and Conflict Level

Pre-service teachers with high levels of neuroticism experience more conflict in student–teacher relationships. Neuroticism causes teachers to feel intense anxiety, tension, and anger, especially in stressful and uncertain situations, resulting in negative consequences in relationships. Pre-service teachers with higher levels of neuroticism tend to experience more conflict in their student–teacher relationships. Neuroticism correlates positively with conflict (r = 0.27), which suggests that teachers who exhibit higher neuroticism may struggle with managing anxiety, tension, and anger in the classroom, leading to more strained interactions with students. Qualitative data show that pre-service teachers with high levels of neuroticism have difficulties in classroom management and tend to have conflict in their interactions with students. The statements of one teacher reflect this situation as follows: “*Sometimes I get frustrated when my students do not take me seriously, and this creates constant tension in our relationship. I find it difficult to assert my authority when I feel more like a big sister or a big brother than a teacher*”. These explanations show that pre-service teachers with high levels of neuroticism experience more conflict in student–teacher relationships, which is related to teachers’ anxiety levels.

#### 3.3.2. High Openness to Experience and Proximity

Pre-service teachers with high levels of openness exhibit more innovative and creative approaches towards students, and this leads to positive effects on student–teacher relationships. This personality trait increases pre-service teachers’ willingness to develop new ideas and experiment with different teaching methods, thus providing students with more varied and richer learning experiences. Similarly, openness to experience was also positively correlated with closeness (r = 0.17), suggesting that pre-service teachers who are more open to new ideas and approaches may create more enriching and creative learning environments, which in turn strengthens their relationships with students.

Qualitative data emphasized that pre-service teachers with high levels of openness used creative activities and innovative methods in their relationships with students. For example, one teacher explained as follows: “*When I try new activities in the classroom, students are more engaged and interested in these innovations. In this way, I feel a stronger bond with them*”. These statements qualitatively support the positive contributions of pre-service teachers’ creative approaches to student–teacher relationships. As a result of openness, creativity and innovation increase student motivation and make classroom relationships more positive.

#### 3.3.3. High Agreeableness and Agreeableness

Pre-service teachers with high levels of agreeableness develop more closeness in student–teacher relationships. Agreeableness increases pre-service teachers’ tendencies to empathize, show understanding, and cooperate, and helps them establish warmer and more sincere relationships with students. Correlation analysis revealed that agreeableness was positively correlated with closeness (r = 0.19), indicating that more empathetic and cooperative pre-service teachers are likely to build warmer, more supportive relationships with students.

In the qualitative interviews, pre-service teachers with high levels of agreeableness reported more positive and supportive relationships with their students. One of these teachers stated, “*I try to be patient and tolerant in my relationships with my students. When I reassure them, they approach me more and are more open to cooperating with me*”. Such statements qualitatively support how agreeableness strengthens student–teacher relationships. Agreeableness stands out as a determining factor for pre-service teachers to establish healthy, trusting relationships with students.

#### 3.3.4. Children’s Temperament Characteristics and Student–Teacher Relations

Children’s temperament characteristics have a significant effect on student–teacher relationships. Researchers found that children’s tendency to react (reactivity) significantly increased their conflict levels with pre-service teachers. Children’s temperament characteristics, particularly reactivity, also have an important role in student–teacher relationships. The data showed a positive correlation between children’s reactivity and conflict (r = 0.16), indicating that more reactive children are likely to experience higher levels of conflict with their teachers. This suggests that managing children’s temperamental tendencies may be critical for fostering harmonious student–teacher relationships.

Qualitative data revealed that pre-service teachers had difficulties in their relationships with reactive children. One teacher described her experiences with reactive children as follows: “*These children overreact to everything, and this creates constant tension in the classroom. It is really difficult to have a healthy relationship with these children*”. Such statements qualitatively confirm that reactive temperaments increase conflict in student–teacher relationships and that teachers have difficulty managing this situation.

This study shows how pre-service teachers’ personality traits and children’s temperaments shape the dynamics of student–teacher relationships. Qualitative data provide a deeper explanation of the different effects of high levels of neuroticism, openness, and agreeableness on teachers’ relationships with students. Neuroticism leads teachers to experience more conflict, while openness and agreeableness lead to more closeness and creativity in student–teacher relationships. Children’s reactive temperament also plays an important role in these relationships. These findings shed light on the multifaceted and complex nature of these relationships.

## 4. Discussion

Preschool education heavily relies on communication and interaction with children, which can significantly impact teachers in this setting. The emotional strain experienced by educators is often characterized by feelings of energy and depletion of emotional resources. Studies indicate a connection between exhaustion and challenges in teacher–child relationships. This highlights the importance of interns receiving support from their peers and supervisors during training situations. Pre-service teachers may react intensely when faced with classroom difficulties, swinging between being overly strict to maintain control or excessively lenient to avoid conflict. This fluctuation between strict and lenient behaviors can hinder the development of a rounded teaching style. Therefore, the support, guidance, reflection, and adjustment opportunities for pre-service teachers are crucial for their professional development and fostering a positive learning atmosphere.

Our first quantitative finding that parents perceive children as less confrontational is consistent with the qualitative interview data. This suggests that pre-service teachers may be more likely to create opportunities to show compassion to less disruptive children in the classroom. A teaching candidate’s professional development progresses faster and more intensively during their teaching practice than at any other stage of the teacher education program [66]. However, it was also seen that while explaining their situation, they could not see this situation as an opportunity to expand their professional knowledge. In addition, the interview findings corroborated the quantitative results that suggested more conflicted student–teacher relationships in the classrooms. For the teachers, the psychological and emotional effort required to interact effectively with children is an often-cited source of stress and fatigue, resulting in their negativity and disconnection from classroom interactions [67]. The emphasis on the dominant role in the process suggests that student–teacher relationships established in the classroom are determined by the temperament characteristics of the children. Moreover, there are statements indicating that pre-service teachers are also influenced by the temperaments of the children in the classrooms where they do their internships. In this context, it can be said that the current finding is supported by the research results [28,68], which point out that teachers’ personal characteristics play an important role in student–teacher relationships. It was determined that the compatibility levels of pre-service teachers explained the situation of the children being perceived as close in the student–teacher relationship. Individuals with high levels in this domain are warm and supportive and, as a result, they tend to develop successful interpersonal relationships at work [69]. These characteristics might contribute to the development of closer relationships with children. Agreeable individuals, defined by their propensity for behaviors that align with the interests of others, readily adopt a team-oriented perspective. In this regard, they usually tend to compromise with others [70,71,72]. This study found a positive correlation between teacher candidates’ compatibility levels and their conceptualizations of close student–teacher relationships. This aligns with existing research highlighting the importance of compatibility in professions demanding high levels of social interaction [73,74]. Generally, the agreeable individual is prone to supporting others’ perspectives and experiences [75]. From this point, adaptive pre-service teachers could create a more harmonic and supported atmosphere, and thus have the potential to develop closer relationships with children.

In addition, it was observed that this situation was negatively affected by the level of neuroticism of the pre-service teachers in the relationship they established with the children when they described them as close. In short, it was seen that the personal characteristics of the teacher candidates have great importance in their perception of closeness. Individuals higher in neuroticism also displayed stronger priming effects for negative targets, but not positive targets [76]. It was found that the pre-service teachers’ characterization of children as confrontational was explained by their neuroticism levels. This is supported by research findings (e.g., [77]), which revealed that individuals with high levels of neuroticism experience some emotional and behavioral difficulties in interpersonal relationships. Finally, for neurotic individuals, tendencies toward generalized negative affective experiences are likely to influence their assessment of their work situation [78]. It is known that neurotic individuals are reactive and show harsh reactions to negative stimuli in their work environment [79]. Children with insecure attachment styles may exhibit heightened reactivity to perceived indifference or emotional distance from caregivers. This heightened sensitivity can create a cycle of escalating negative interactions, potentially contributing to the development of neurotic tendencies. Conversely, secure attachment styles are characterized by a sense of trust and emotional security. This allows children to develop healthier coping mechanisms for managing negative stimuli and meeting their emotional needs. Consequently, secure attachment styles are associated with less frequent and less intense neurotic reactions [80,81].

There was no effect of the children’s gender on the relationship between students and teachers. This finding did not align with research findings that showed a gender effect on student–teacher relationships, such as Furrer and Skinner [19]. This was supported by other studies like Murray, Waas, and Murray [82]. Furthermore, the reactive temperament traits of the children were found to influence how pre-service teachers perceived them as confrontational. This discovery was consistent with research indicating that children with a confrontational temperament can impact student–teacher dynamics [83,84,85]. The temperament characteristics of children were also corroborated by research showing associations with their issues [86]. In summary, this situation can influence how negative behaviors exhibited by the children are perceived and evaluated by pre-service teachers.

## 5. Conclusions and Future Research

The perspectives of preschool pre-service teachers on student–teacher relationships were examined in light of quantitative and qualitative research methods. As far as could be determined (as far as could be found in the relevant literature), no research has directly examined the predictive power of teacher personality traits on the student–teacher relationship. In this study, it was determined that the pre-service teachers’ evaluations of the student–teacher relationship (intimacy and conflict) obtained through self-reports were explained by the personality traits of the teachers as well as by the temperament characteristics of the children. It was revealed that the pre-service teachers with a high level of neuroticism evaluated the children as confrontational. This situation suggested the potential of a teacher’s personality traits in playing an important role in their evaluations of children. As in all other studies that revealed the explanatory effect of children’s temperament characteristics on the student–teacher relationship in general, as a result of this research, it was determined that the children’s reactive temperament characteristics predicted confrontational student–teacher relationships.

Interviews with pre-service teachers revealed a tendency to prioritize individual student differences and temperament when building supportive student–teacher relationships. This suggests a potential disconnect in fostering positive interactions within the classroom. This situation brought about the thought that they are not able to develop an understanding towards developing a trust-based relationship with children, which is the basis of the supportive student–teacher relationship.

## 6. Limitations

Our study has several limitations. Firstly, the sample of pre-service teachers is limited due to being a convenience sample and may not be fully representative of a larger population of pre-service teachers. Future research should use a larger and more diverse sample to ensure that the findings are more generalizable. Secondly, the cross-sectional nature of the data limited our ability to observe changes in pre-service teachers’ perceptions and behaviors over time. Longitudinal studies would be useful to understand how pre-service teachers’ perspectives and relationships with students develop throughout their education and early careers. Finally, we measured only a few aspects of teacher personality traits and children’s temperaments, which may not fully reflect the complexity of student–teacher relationships. Future studies should include a broader range of personality traits and temperament dimensions to provide a more comprehensive understanding.

## 7. Implications

Despite these limitations, this study has several significant implications. Theoretically, our study expands the discourse by incorporating the role of teacher personality traits in the student–teacher relationship, an area previously underexplored. The findings highlight the importance of considering teacher personality traits, such as neuroticism, in understanding their interactions with students. Practically, these insights can inform teacher training programs to better prepare pre-service teachers for the emotional and psychological challenges of the classroom.

Although a correlational relationship was found between teacher personality traits and the quality of the teacher–student relationship, no predictive relationship emerged in the regression analysis. This suggests that while personality traits may be associated with relational dynamics, they do not necessarily act as strong predictors of relationship outcomes. Instead, it is possible that more professional identity-related variables, such as teacher self-efficacy or professional competencies, may play a more significant role in shaping the quality of teacher–student relationships. Therefore, future research should focus on exploring these professional characteristics in both pre-service and in-service teachers to better understand their impact on teacher–student interactions. Prioritizing such investigations may provide deeper insights into how the professional development of teachers contributes to the cultivation of positive and effective teacher–student relationships.

From a practical standpoint, our findings suggest that teacher education programs should emphasize the development of self-awareness and emotional regulation skills among pre-service teachers. By increasing pre-service teachers’ awareness of their personality traits and how these traits influence their interactions with students, teacher education programs can help them develop more effective and supportive student–teacher relationships. Providing pre-service teachers with strategies to manage stress and emotional challenges can also enhance their ability to create positive learning environments.

In addition, our findings suggest the need for tailored support and resources at practicum schools to address the individual differences among pre-service teachers. By offering targeted interventions and support based on the unique characteristics of each teacher, practicum schools can foster a more supportive and effective learning environment for both teachers and students. This approach can ultimately contribute to better educational outcomes and a more positive classroom climate.

Lastly, the results indicate the importance of ongoing professional development and support for pre-service teachers as they transition into their teaching careers. By continuing to provide resources and guidance on emotional and psychological well-being, teacher education programs can ensure that new teachers are well-equipped to navigate the challenges of the classroom and build strong, trust-based relationships with their students.

Building on the existing suggestions, it is important to emphasize that teacher education programs should prioritize the development of self-awareness and self-regulation skills among pre-service teachers. Understanding how their personality traits influence their perceptions of students is crucial for fostering more objective and supportive teacher–student relationships. Programs should include training that helps pre-service teachers recognize and manage potential biases arising from their personality traits, as well as structured activities that enhance their self-awareness. Additionally, ongoing feedback and support during their training can help them continuously refine these skills, ultimately leading to more effective classroom management and positive learning environments. Integrating these elements into teacher training will better prepare pre-service teachers for the emotional and psychological challenges they will face in their teaching careers. Faculties should offer more opportunities for teamwork to enhance teaching candidates’ collaborative skills. Similarly, faculties should provide psychological counseling services to improve the mental health status of teaching candidates.

## Figures and Tables

**Table 1:** Distribution of the interviewed teachers according to their personality traits.

Personality Characteristics	Pre-Service Teachers
High level of neuroticism	6
High level of openness	6
High level of agreeableness	6

**Table 2:** Correlation coefficients and descriptive statistics for the variables of the pre-service teachers.

Variables	1	2	3	4	5	6	7	8	9	10	Mean	SD
Closeness	-										3.00	1.609
Conflict	−0.19 **										1.52	0.501
Neuroticism	0.06	0.27 **									3.15	1.061
Conscientiousness	0.07	−0.10	0.15								2.85	0.582
Openness to experience	0.17 *	0.09	0.14	0.03							2.57	0.956
Extraversion	0.05	0.10	0.00	0.02	0.09						3.17	0.936
Agreeableness	0.19 **	0.04	0.07	0.15	0.05	−0.05					3.63	0.597
Reactivity	−0.18	0.16 *	−0.09	0.14	0.04	−0.09	0.01				2.83	1.269
Approach	0.11	0.08	−0.09	0.00	0.05	0.05	0.10	−0.02			3.97	1.115
Persistence	0.02	0.13	0.03	0.07	0.02	0.04	0.07	−0.08	0.12		4.05	1.300

Note. * *p* < 0.05, ** *p* < 0.01.

**Table 3:** Multiple linear regression model for the pre-service teachers.

Variables		Closeness				Conflict		
	SHB	β	t	*p*	SHB	β	t	*p*
Constant	3.279		0.968	0.06	4.47		0.865	0.389
Child’s gender	0.215	0.047	0.511	0.117	0.293	−0.041	−0.447	0.656
Neuroticism	0.45	−0.524	−1.296	0.542	0.613	0.682	1.685	0.095
Conscientiousness	0.804	0.653	1.646	0.781	1.095	−0.719	−1.814	0.072
Openness to experience	0.402	0.406	1.245	0.978	0.548	−0.299	−0.918	0.36
Extraversion	0.338	−0.272	−1.589	0.871	0.554	0.495	1.606	0.545
Agreeableness	0.406	−0.168	−0.52	0.907	0.46	0.212	1.238	0.218
Reactivity	0.329	0.261	0.736	0.275	0.449	0.593	1.827	0.41
Approach	0.358	0.112	0.331	0.447	0.489	−0.19	−0.561	0.576
Persistence	0.282	0.154	0.494	0.819	0.385	−0.107	−0.345	0.731

## Data Availability

The raw data collected by the scales are available upon request to the corresponding author.

## References

[1] Pianta R.C. (2001). Student-Teacher Relationship Scale: Professional Manual.

[2] Bowlby J. (1982). Attachment and Loss: Vol 1. Attachment.

[3] Ainsworth M.S. (1989). Attachments beyond infancy. Am. Psychol..

[4] Sroufe L.A., Carlson E.A., Levy A.K., Egeland B. (1999). Implications of attachment theory for developmental psychopathology. Dev. Psychopathol..

[5] Pianta R.C., Steinberg M.S., Rollins K.B. (1995). The first two years of school: Student-teacher relationships and deflections in children’s classroom adjustment. Dev. Psychopathol..

[6] O’Connor E., McCartney K. (2007). Attachment and cognitive skills: An investigation of mediating mechanisms. J. Appl. Dev. Psychol..

[7] Pianta R.C., Hamre B.K., Allen J.P. (2012). Teacher-student relationships and engagement: Conceptualizing, measuring, and improving the capacity of classroom interactions. Handbook of Research on Student Engagement.

[8] Howes C., Phillipsen L.C., Peisner-Feinberg E. (2000). The Consistency of Perceived Teacher–Child Relationships between Preschool and Kindergarten. J. Sch. Psychol..

[9] Hughes J.N., Cavell T.A., Wilson V. (2001). Further support for the developmental significance of the quality of the teacher–student relationship. J. Sch. Psychol..

[10] Mantzicopoulos P. (2005). Conflictual relationships between kindergarten children and their teachers: Associations with child and classroom context variables. J. Sch. Psychol..

[11] Alamos P., Williford A.P. (2019). Exploring dyadic teacher–child interactions, emotional security, and task engagement in preschool children displaying externalizing behaviors. Soc. Dev..

[12] Ryan R.M., Deci E.L. (2000). Intrinsic and extrinsic motivations: Classic definitions and new directions. Contemp. Educ. Psychol..

[13] Birch S.H., Ladd G.W. (1997). The teacher-child relationship and children’s early school adjustment. J. Sch. Psychol..

[14] Hughes J.N., Wu J.Y., Kwok O.M., Villarreal V., Johnson A.Y. (2012). Indirect effects of child reports of teacher–student relationship on achievement. J. Educ. Psychol..

[15] Granic I., Patterson G.R. (2006). Toward a comprehensive model of antisocial development: A dynamic systems approach. Psychol. Rev..

[16] Doumen S., Verschueren K., Buyse E., Germeijs V., Luyckx K., Soenens B. (2008). Reciprocal relations between teacher–child conflict and aggressive behavior in kindergarten: A three-wave longitudinal study. J. Clin. Child Adolesc. Psychol..

[17] Hartz K., Williford A.P., Koomen H.M. (2017). Teachers’ perceptions of teacher–child relationships: Links with children’s observed interactions. Early Educ. Dev..

[18] Hughes J.N., Kwok O.M. (2006). Classroom engagement mediates the effect of teacher–student support on elementary students’ peer acceptance: A prospective analysis. J. Sch. Psychol..

[19] Furrer C., Skinner E. (2003). Sense of relatedness as a factor in children’s academic engagement and performance. J. Educ. Psychol..

[20] Rudasill K.M., Rimm-Kaufman S.E. (2009). Teacher–child relationship quality: The roles of child temperament and teacher–child interactions. Early Child. Res. Q..

[21] Graves S.L., Howes C. (2011). Ethnic differences in social-emotional development in preschool: The impact of teacher-child relationships and classroom quality. Sch. Psychol. Q..

[22] Sointu E.T., Savolainen H., Lappalainen K., Lambert M.C. (2017). Longitudinal associations of student–teacher relationships and behavioral and emotional strengths on academic achievement. Educ. Psychol..

[23] Cadima J., Verschueren K., Leal T., Guedes C. (2016). Classroom interactions, dyadic teacher–child relationships, and self–regulation in socially disadvantaged young children. J. Abnorm. Child Psychol..

[24] Dede Yildirim E., Frosch C.A., Santos A.J., Veríssimo M., Bub K., Vaughn B.E. (2024). Antecedents to and outcomes associated with teacher–child relationship perceptions in early childhood: Further evidence for child-driven effects. Child Dev..

[25] Paes T.M., Duncan R., Purpura D.J., Schmitt S.A. (2023). The relations between teacher-child relationships in preschool and children’s outcomes in kindergarten. J. Appl. Dev. Psychol..

[26] Šumatić M., Malmberg L.E., Gregoriadis A., Grammatikopoulos V., Zachopoulou E. (2023). Child, teacher and preschool characteristics and child-teacher relationships in Greek preschools. Early Child. Res. Q..

[27] Embacher E.M., Smidt W. (2023). Associations between teachers’ professional competencies and the quality of interactions and relationships in preschool: Findings from Austria. Front. Psychol..

[28] Wang Y., Pan B., Yu Z., Song Z. (2024). The relationship between preschool teacher trait mindfulness and teacher-child relationship quality: The chain mediating role of emotional intelligence and empathy. Curr. Psychol..

[29] Hughes J.N., Luo W., Kwok O.-M., Loyd L.K. (2008). Teacher-student support, effortful engagement, and achievement: A 3-year longitudinal study. J. Educ. Psychol..

[30] Maldonado-Carreno C., Votruba-Drzal E. (2011). Student-teacher relationships and the development of academic and behavioral skills during elementary school: A within-and between-child analysis. Child Dev..

[31] Sabol T.J., Pianta R.C. (2012). Recent trends in research on teacher–child relationships. Attach. Hum. Dev..

[32] Jamil F.M., Downer J.T., Pianta R.C. (2012). Association of pre-service teachers’ performance, personality, and beliefs with teacher self-efficacy at program completion. Teach. Educ. Q..

[33] Klassen R.M., Tze V.M. (2014). Teachers’ self-efficacy, personality, and teaching effectiveness: A meta-analysis. Educ. Res. Rev..

[34] Bullock A., Coplan R.J., Bosacki S. (2015). Exploring links between early childhood educators’ psychological characteristics and classroom management self-efficacy beliefs. Can. J. Behav. Sci./Rev. Can. Des Sci. Du Comport..

[35] Rimm-Kaufman S.E., Hamre B.K. (2010). The role of psychological and developmental science in efforts to improve teacher quality. Teach. Coll. Rec..

[36] Decker L.E., Rimm-Kaufman S.E. (2008). Personality characteristics and teacher beliefs among pre-service teachers. Teach. Educ. Q..

[37] Fajet W., Bello M., Leftwich S.A., Mesler J.L., Shaver A.N. (2005). Pre-service teachers’ perceptions in beginning education classes. Teach. Teach. Educ..

[38] De Jong R., Mainhard T., Van Tartwijk J., Veldman I., Verloop N., Wubbels T. (2014). How pre-service teachers’ personality traits, self-efficacy, and discipline strategies contribute to the teacher–student relationship. Br. J. Educ. Psychol..

[39] Downer J.T., Goble P., Myers S.S., Pianta R.C. (2016). Student-teacher racial/ethnic match within pre-kindergarten classrooms and children’s early school adjustment. Early Child. Res. Q..

[40] Gilliam W.S., Maupin A.N., Reyes C.R., Accavitti M., Shic F. (2016). Do early educators’ implicit biases regarding sex and race relate to behavior expectations and recommendations of preschool expulsions and suspensions?. Yale Univ. Child Study Cent..

[41] Corbin C.M., Alamos P., Lowenstein A.E., Downer J.T., Brown J.L. (2019). The role of teacher-student relationships in predicting teachers’ personal accomplishment and emotional exhaustion. J. Sch. Psychol..

[42] Murray C., Murray K.M. (2004). Child level correlates of teacher–student relationships: An examination of demographic characteristics, academic orientations, and behavioral orientations. Psychol. Sch..

[43] Pramling Samuelsson I. (2011). Why we should begin early with ESD: The role of early childhood education. Int. J. Early Child..

[44] Pramling Samuelsson I., Asplund Carlsson M. (2008). The playing learning child: Towards a pedagogy of early childhood. Scand. J. Educ..

[45] Johansson E., Pramling Samuelsson I. (2009). To weave together-play and learning in early childhood education. J. Aust. Res. Early Child. Educ..

[46] Howes C., James J., Ritchie S. (2003). Pathways to effective teaching. Early Child. Res. Q..

[47] Alvestad M., Röthle M. (2007). Educational Forums: Frames for development of professional learning. A project in early childhood education in Norway. Eur. Early Child. Educ. Res. J..

[48] Gourgiotou E. (2017). Trainee teachers’ collaborative and reflective practicum in kindergarten classrooms in Greece: A case study approach. Educ. Rev..

[49] Pierce K.M. (2007). Betwixt and between: Liminality in beginning teaching. New Educ..

[50] Zeichner K. (2010). New epistemologies in teacher education. Rethinking the connections between campus courses and practical experiences in teacher education at the university. Interuniv. J. Teach. Educ..

[51] Baum A.C., Korth B.B. (2013). Preparing Classroom Teachers to Be Cooperating Teachers: A Report of Current Efforts, Beliefs, Challenges, and Associated Recommendations. J. Early Child. Teach. Educ..

[52] Retallick M.S., Miller G. (2010). Teacher Preparation in Career and Technical Education: A Model for Developing and Researching Early Field Experiences. J. Career Tech. Educ..

[53] Creswell J.W. (2005). Educational Research: Planning, Conducting, and Evaluating Quantitative and Qualitative Research.

[54] Creswell J.W., Plano Clark V.L., Gutmann M., ve Hanson W., Tashakkori A., ve Teddlie C. (2003). Advanced mixed methods research designs. Handbook of Mixed Methods in Social and Behavioral Research.

[55] Tashakkori A., Creswell J.W. (2007). The new era of mixed methods. J. Mix. Methods Res..

[56] Ası D.Ş., Karabay Ş.O. (2018). Öğrenci-öğretmen ilişki ölçeği-kısa formunun Türkçe’ye uyarlanması. Ege Eğitim Derg..

[57] Prior M., Sanson A., Carroll R., Oberklaid F. (1989). Social class differences in temperament ratings by mothers of preschool children. Merrill-Palmer Q..

[58] Yağmurlu B., Sanson A. (2009). Parenting and temperament as predictors of prosocial behaviour in Australian and Turkish Australian children. Aust. J. Psychol..

[59] Rammstedt B., John O.P. (2007). Measuring personality in one minute or less: A 10-item short version of the Big Five Inventory in English and German. J. Res. Personal..

[60] Horzum M.B., Ayas T., Padir M.A. (2017). Beş Faktör Kişilik Ölçeğinin Türk Kültürüne Uyarlanmasi. Sak. Univ. J. Educ..

[61] Hahs-Vaughn D.L., Lomax R.G. (2020). An Introduction to Statistical Concepts.

[62] Merriam S.B. (2009). Qualitative Research: A Guide to Design and Implementation.

[63] Bernard H.R., Ryan G.W. (2010). Analyzing Qualitative Data: Systematic Approaches.

[64] Braun V., Clarke V. (2006). Using thematic analysis in psychology. Qual. Res. Psychol..

[65] Creswell J.W., Bütün M., ve Demir S.B. (2014). Nitel Araştırma Yöntemleri.

[66] Caires S., Almeida L., Vieira D. (2012). Becoming a teacher: Student teachers’ experiences and perceptions about teaching practice. Eur. J. Teach. Educ..

[67] Jennings P.A. (2015). Early childhood teachers’ well-being, mindfulness, and self-compassion about classroom quality and attitudes towards challenging students. Mindfulness.

[68] Embacher E.M., Zöggeler-Burkhardt L., Smidt W. (2023). Closeness and conflict in teacher-child relationships in preschool: The role of child personality types. Early Child Dev. Care.

[69] Organ D.W., Lingl A. (1995). Personality, satisfaction, and organizational citizenship behavior. J. Soc. Psychol..

[70] Ann B.Y., Yang C.C. (2012). The Moderating Role of Personality Traits on Emotional Intelligence and Conflict Management Styles. Psychol. Rep..

[71] Ejaz S.S., Iqbal F., Ara A. (2012). Relationship among personality traits and conflict handling styles of call center representatives and appraisal of existing service model. Int. J. Psychol. Stud..

[72] Messarra L.C., Karkoulian S., El-Kassar A.N. (2016). Conflict resolution styles and personality: The moderating effect of generation X and Y in a non-Western context. Int. J. Product. Perform. Manag..

[73] Kim L.E., Dar-Nimrod I., MacCann C. (2018). Teacher personality and teacher effectiveness in secondary school: Personality predicts teacher support and student self-efficacy but not academic achievement. J. Educ. Psychol..

[74] Hirshberg M.J., Flook L., Enright R.D., Davidson R.J. (2020). Integrating mindfulness and connection practices into preservice teacher education improves classroom practices. Learn. Instr..

[75] Neuman G.A., Wagner S.H., Christiansen N.D. (1999). The Relationship between Work-Team Personality Composition and the Job Performance of Teams. Group Organ. Manag..

[76] Robinson M.D., Ode S., Moeller S.K., Goetz P.W. (2007). Neuroticism and affective priming: Evidence for a neuroticism-linked negative schema. Personal. Individ. Differ..

[77] Robinson M.D., Meier B.P. (2005). Rotten to the core: Neuroticism and implicit evaluations of the self. Self Identity.

[78] Bruk-Lee V., Khoury H.A., Nixon A.E., Goh A., Spector P.E. (2009). Replicating and Extending Past Personality/Job Satisfaction Meta-Analyses. Hum. Perform..

[79] Ilies R., Judge T.A. (2004). An experience-sampling measure of job satisfaction and its relationships with affectivity, mood at work, job beliefs, and general job satisfaction. Eur. J. Work Organ. Psychol..

[80] Cervera-Solís V.I., Muñoz Suárez M.A., Cortés Sotres J.F., Hernández Lagunas J.O., Díaz-Anzaldúa A. (2022). Attachment styles predict personality traits according to a pilot study of patients with anxiety and mood disorders. Salud Ment..

[81] Fan P. (2023). The Relationship between Insecure Attachment and Personality. J. Educ. Humanit. Soc. Sci..

[82] Murray C., Murray K.M., Waas G.A. (2008). Child and teacher reports of teacher–student relationships: Concordance of perspectives and associations with school adjustment in urban kindergarten classrooms. J. Appl. Dev. Psychol..

[83] De Schipper J.C., Tavecchio L.W., Van IJzendoorn M.H., Van Zeijl J. (2004). Goodness-of-fit in center day care: Relations of temperament, stability, and quality of care with the child’s adjustment. Early Child. Res. Q..

[84] Griggs M.S., Gagnon S.G., Huelsman T.J., Kidder-Ashley P., Ballard M. (2009). Student–teacher relationships matter: Moderating influences between temperament and preschool social competence. Psychol. Sch..

[85] Valiente C., Swanson J., Lemery-Chalfant K. (2012). Kindergartners’ temperament, classroom engagement, and student–teacher relationship: Moderation by effortful control. Soc. Dev..

[86] Delgado B., Carrasco M.A., González-Peña P., Holgado-Tello F.P. (2018). Temperament and behavioral problems in young children: The protective role of extraversion and effortful control. J. Child Fam. Stud..

